# A prospective study on a cohort of horses and ponies selected for participation in the European Eventing Championship: reasons for withdrawal and predictive value of fitness tests

**DOI:** 10.1186/1746-6148-9-182

**Published:** 2013-09-13

**Authors:** Carolien C B M Munsters, Jan van den Broek, Emile Welling, René van Weeren, Marianne M Sloet van Oldruitenborgh-Oosterbaan

**Affiliations:** 1 Department of Equine Sciences, Faculty of Veterinary Medicine, Utrecht University, Yalelaan 114, 3584 CM Utrecht, the Netherlands; 2 Moxie Sport Analysis & Coaching, Raam 107, 5403 TH Uden, the Netherlands; 3 Department of Farm Animal Health, Utrecht University, Yalelaan 7, 3584 CL Utrecht, the Netherlands; 4 Dutch National Equestrian Federation, de Beek 125, 3852 PL Ermelo, the Netherlands

**Keywords:** Horse, Eventing, Exercise, Fitness, Monitoring, Training

## Abstract

**Background:**

Eventing is generally recognized as a challenging equestrian discipline and wastage figures for this discipline are relatively high. There is a need for information that provides insight into the causes of wastage and withdrawal from competition, for animal welfare and economic reasons. The aim of the present investigation was to conduct a prospective study following the entire national selection of event horses (n = 20) and ponies (n = 9) in the Netherlands that prepared for the European Championship in 2010 (ponies) and 2011 (horses), noting causes of withdrawal and monitoring fitness using standardized exercise tests (SETs), with heart rate (HR; beats/min), speed (V; m/s) and plasma lactate concentrations (LA; mmol/L) as measured parameters.

**Results:**

In SET-I, performed at the beginning of the season, horses (n = 17) had a mean V_LA4_ (V at LA 4 mmol/L) of 10.3 ± 0.4 m/s with a mean V_200_ (V at 200 beats/min) of 11.4 ± 0.8 m/s and ponies (n = 9) a mean V_LA4_ of 7.8 ± 0.9 m/s and V_200_ of 9.6 ± 0.7 m/s. Before SET-II, performed six weeks before the European Championship, 16/20 horses and 6/9 ponies were withdrawn. The most common reason for withdrawal was locomotor injury (9/16 horses, 4/6 ponies; P < 0.001 and P = 0.011, respectively). Other reasons included an animal ‘not meeting the competition criteria’ (4/16 horses, 2/6 ponies) and being sold (3/16 horses). Animals were divided on the basis of V_LA4_ and recovery-HR during SET-I into good and average performers. Average performers were significantly more likely to be injured (50.0%) than good performers (0%, P = 0.05). In a subpopulation of ten horses, in which all condition training sessions were evaluated for HR and speed, HR_peak_ was significantly lower in horses that stayed sound (186 ± 9 beats/min) compared with horses withdrawn from training and competition because of injury (201 ± 5 beats/min; P = 0.016).

**Conclusions:**

Of the national selection, 45% of all animals were unavailable for the European Championship because of locomotor injuries. Field tests were useful in assessing the potential injury risk, as individuals with better fitness indices (good performers) were less likely to become injured than average performers. Furthermore, monitoring of training sessions showed predictive value for future injuries, as horses withdrawn because of injury later on showed already higher peak HRs during condition training than horses that stayed sound. Therefore the increase in peak HR seemed to precede visible lameness in a horse.

## Background

Eventing is generally recognized as a challenging equestrian discipline for both rider and horse. The European Championships for eventing horses and ponies are competitions at the highest level, set by the Federation Equestrian International (FEI) and comprising a three day event with three distinct tests: dressage, cross-country, and show jumping. Equine wastage figures for this discipline during training as well as in competition are relatively high [1,2]. Therefore, there is a need, for both animal welfare and economic reasons, to develop information that provides insight into the causes of such wastage and consequent withdrawal from competition.

The fitness of event horses at different competitive levels up to Concours Complet International 2-star (CCI**) has been assessed using standardized exercise tests (SETs) that measure heart rate (HR), speed (V), and plasma lactate concentration (LA) [3-6]. In horses, the European Championship is ranked at the CCI***(3-star) level but is generally considered to be equal to a 4-star event [7]. To the authors’ knowledge, physiological parameters of event horses and ponies at the European Championship level have not been reported to date. The cross-country phase in horses at the CCI*** level comprises a distance up to 6270 m with 40 jumping efforts which should be completed at 570 m/min (9.5 m/s)^1^. During the cross-country phase, HRs of ~75% of maximum HR and LA in >4 mmol/L indicate anaerobic work effort by the horses [3,8]. Studies of event horses competing at the CCI*** level have shown that horses achieved HRs of 171 ± 19 beats/min and LA concentrations of 19.1 ± 4.2 mmol/L [8,9] or 10.2 ± 3.1 mmol/L [10] during the cross-country phase. Event horses competing at the CCI**** level achieved HRs of 188 ± 11 beats/min and LA concentrations of 22.4 ± 11 mmol/L during the cross-country phase [7]. In all three studies, the HRs and LA concentrations of individual animals achieved during the cross-country phase ranged widely and represented individual differences in fitness, training level, and/or maximal heart rate, but also reflected differences in types of exercise (intensity, duration) during sampling, as well as differences in when the sample was collected, versus what the horse was being asked to do and its individual capability [7-10].

In ponies, the European Championship is ranked at the CCI** level, comprising a cross-country phase with a distance up to 4160 m with 30 jumping efforts which should be completed at 520 m/min (8.7 m/s).^1^ No data are available on the intensity of event competitions for ponies and there is, in general, very little information regarding exercise testing in ponies [11,12].

The Code of Conduct of the FEI states that the frequency of competitions and any other risk factors should be examined carefully to minimize injuries.^1^ There is therefore a need to closely monitor fitness, workload, and injuries in event horses because such information might contribute to understanding the effects of training methods and might help reduce injuries. It has been shown that several calculated fitness parameters of Standardbred and Warmblood horses can be used to optimize training and, hence, indirectly affect performance or incidence of injuries [4,13-15].

The aim of the present investigation was to conduct a prospective study of the entire national selection of event horses and ponies in the Netherlands that prepared for the European Championship in 2010 (ponies) and 2011 (horses), noting causes of withdrawal and monitoring fitness using SETs. It was hypothesized that differences in fitness level of horses and ponies might contribute to injury occurrence. Additionally, the predictive value of a field SET, carried out at the start of the competition season, was assessed regarding subsequent injuries. Furthermore, in a subpopulation of horses, all condition-training sessions were also evaluated in relation to injury occurrence.

## Methods

### Study design

This prospective cohort study evaluated the fitness and causes for withdrawal in the entire national selection of event horses and event ponies in the Netherlands during preparations for the European Championships in 2010–2011. In setting up the study and related report, the STROBE guidelines [16] were followed whenever possible.

The cohort consisted of the entire national selection of twenty event horses and nine ponies in the Netherlands engaged in preparations for the European Championship in 2010 (ponies, at CCI** level held in Bishop Burton, UK) and 2011 (horses, at CCI*** level held in Luhmühlen, Germany). The fitness of each animal was evaluated using a SET at the beginning of the competition season (March, 2010 and March, 2011 for ponies and horses, respectively; SET-I) and six weeks before the European Championships (SET-II; eleven weeks after SET-I). During this preparation period, causes for withdrawal were noted and injuries assessed by the team veterinarian. Furthermore, in a subpopulation of ten horses, HR and V data from all condition training sessions at home were collected and, in horses, the number, level, and date of competitions also noted.

The Animal Ethics Committee of Utrecht University concluded that the proposed study did not need ethical approval, as it did not qualify as an animal experiment under Dutch law, but individual horse owner’s consent was obtained for all horses and ponies participating in this study.

### Study subjects

All animals were assigned to the national selection at the beginning of the competition year by the Dutch National Equestrian Federation (KNHS) on the basis of their performance during the preceding competition year. The animal’s performance had to meet the KNHS’s minimum eligibility requirements for eventing^2^; for horses, being qualified at the CIC/CCI*** level with <80.5 points, and for ponies, being qualified at Novice level. Therefore, the animals involved in this cohort study were selected using two eligibility criteria, being assigned by the KNHS to the national selection and housed in the Netherlands.

Twenty horses (16 geldings and 4 mares) and nine ponies (5 geldings and 4 mares) were tested and found to possess various levels of fitness and experience. All horses and ponies were trained prior to SET-I (and during the preceding competition year) and between SETs for CIC/CCI*** or CCI**** events (horses) or for Novice/CIC* or CIC** events (ponies); all animals were ridden by their usual rider during SETs and training sessions. Animals were housed in individual stables, provided water *ad libitum*, and fed an individual diet.

### Equipment

During SETs and condition training sessions, animals were equipped with an HR-monitor, Polar-RS800 that recorded HR (beats/min) at 12 Hz and speed (m/s) simultaneously with an integral GPS system (Polar Electro Oy, Oulo, Finland) [17]. The HR device consisted of two soft cotton-like transmitters containing electrodes, with one electrode placed under the girth behind the left elbow and the other placed under the saddle at the withers [18]. To optimize contact, electrodes and skin were wetted with water.

During each SET, plasma LA (mmol/L) was measured immediately with a portable hand-held lactate measurement device (Lactate Pro®, Arkray, Inc., KDK Co., Ltd., Kyoto, Japan) [19]. From each horse, single samples were taken from the jugular vein at rest within 1 min after each incremental step of the SET. As lactate concentrations <0.8 mmol/L were under the detection limit, these values were all set at 0.8 mmol/L.

### Fitness assessment

The fitness level of horses and ponies was evaluated by SETs performed at the beginning (SET-I) of the competition season and six weeks before the European Championship (SET-II). During SETs, HR, V, and LA were measured. Ambient temperature (°C) and relative humidity (RH; %) were continuously registered with a weather station (Silva-ADC-pro, Silva Sweden AB, Sollentuna, Sweden). In the effort to limit bias, all SETs for horses and ponies were performed on a 1000-m racetrack with sand footing and all SETs comprised a warm-up walk (4 min, 400 m), trot (4 min, 600 m), and four incremental exercise steps. After each exercise step, animals were slowed down, walked for 5 min, and within 1 min of the walk, horses were briefly stopped and blood samples collected.

In horses, the SET consisted of four consecutive 1000-m gallops at speeds aimed at 6.7, 8.3, 10.0, and 11.7 m/s (or at a horse’s individual maximal speed) [5,6]. Blood samples were taken after each exercise step and again after 10 min of a cool-down walk to measure the horse’s recovery. During the SET, riders controlled the speed using a GPS system. In ponies, warm-up and SET were the same as in horses and consisted of four consecutive 1000-m gallops, but SET-I was performed on a different racetrack (2 × 500 m in a straight line, turning at the end). As pony riders had difficulties riding at a predetermined speed, they rode at a predetermined HR, aiming for 160, 170, and 180 beats/min and a final step at each horse’s individual maximal speed [20].

### Withdrawal of animals

Causes for withdrawal of animals before the European Championships were noted during the period between SET-I and SET-II. All injuries were assessed and determined by the team veterinarian. Animals were assigned to the national selection based on the KNHS selection criteria and also had to qualify at this competition level during preparation for the European Championships. If this was not possible, horses could not meet the competition criteria necessary to participate at the European Championship.

### Condition training sessions

Condition training sessions were chosen with the intent of evaluating exercise sessions employed by elite event riders to prepare their horses for CIC/CCI*** or CCI**** events. Training sessions for horses being prepared for competition were typically intense exercise sessions. In a subpopulation of ten horses randomly chosen from the entire national selection of twenty event horses, HR and V data from all condition training sessions at home were collected in the period between SET-I and SET-II. The number of condition training sessions, frequency, and condition training session versus competition ratio were recorded. For each horse, each condition training session was evaluated for the number of training bouts per session, mean duration of these bouts, and mean and peak HR and V of each bout.

### Data processing

After completion of the SETs, the mean HR and V of each exercise step for each horse and pony were calculated by averaging HRs and Vs from the last minute of each exercise step [4,13]. The relationships of LA to HR and to V were determined by plotting HR and V against LA as an exponential regression curve. The V_LA2_ and V_LA4_ (V at LA of 2 and 4 mmol/L, respectively) and HR_LA2_ and HR_LA4_ (HR at LA of 2 and 4 mmol/L, respectively) were determined by interpolation. The relationship between HR and V was approximated by a linear regression curve to determine V at a HR of 140, 170, and 200 beats/min (V_140_, V_170_, and V_200_, respectively). Recovery HRs after 5 and 10 min (HR_rec5_ and HR_rec10_, respectively) after the last exercise step were also obtained.

According to the studies of Bitschnau et al. [4] and Couroucé et al. [13] Warmblood sport horses and Standardbred trotters can be divided into performance groups based on whether the individual V_LA4_ and HR recovery are above or below the median. In the present study, event horses and ponies were, according to the above protocol, divided into average or good performers (AP or GP, respectively) depending on whether the individual V_LA4_ and HR recovery was above or below the median V_LA4_ and HR recovery values of SET-I [4,10].

### Statistics

All data are presented as mean ± sd or percentages. In the case of the general linear model and the *t*-test the normality assumption was checked with the normal probability plot of the residual. If the normality assumption did not hold, the data were log-transformed.

The predictive value of the SET-I data was evaluated for forecasting an injury later on by analysis of horse and pony data using a logistic-regression with injury as a dependent variable, and age, sex, and horse/pony interactions as independent variables. The significance of the relationship of these fixed-effects to injury occurrence was determined by likelihood-ratio-tests. The Fisher-exact-test was used to analyze whether horses or ponies were significantly more likely to be injured and/or differently distributed between the performance groups and whether there was a difference between the performance groups with respect to injury occurrence (P < 0.05). The sex and age variables were considered to be potential confounders and horse/pony was considered to be an effect modifier of SET-I data.

The presence of differences in individual values of V_LA2_, V_LA4_, HR_LA2_, and HR_LA4_ between SETs was evaluated using partial-correlations with Fisher’s *z*-transformation as the confidence interval used. Weather conditions, including ambient temperature and relative humidity, were evaluated using *t*-tests between SETs (P < 0.05).

In the subpopulation of ten horses, mean and peak HR and V data of condition training sessions were analyzed using a general linear-model with injury as a fixed-effect and horse as a random-effect. Chi-square tests were used to evaluate reasons for withdrawal from the European Championship and competitions data (P < 0.05). Computations were performed with R (R: A Language and Environment for Statistical Computing, Vienna, Austria 2010) and SPSS 17.0 (IBM Corp., Amonk, NY, USA).

## Results

The initial level and competition program of the twenty event horses (12.1 ± 2.4 yr) and nine event ponies (10.9 ± 2.9 yr) in the preparation for the European Championships are described in Table 1. For evaluation of SET-I, some horses were not included in the final statistical evaluation because of incomplete SET-I data (n = 3).

**Table 1:** Competition level, frequency, and training of event horses and ponies of the entire national selection

	**Horses**	**Ponies**	**Total**
	**n**	**n**	**n**
Total animals (n)	20	9	29
**Competition level at beginning of season**	**20**	**9**	**29**
Competed at level comparable to EC	11	6	17
Never before competed at a level comparable to EC	9	3	12
**Competitions during preparation phase**			
Total number	4.0 ± 1.8	N.A.	
Competition per level:			
CCI*** or CCI****	2.3 ± 1.8	N.A.	
CIC***	0.9 ± 1.1	N.A.	
CIC** or CCI**	0.7 ± 1.2	N.A.	
Number of training weeks before first competition*	6.7 ± 4.1	N.A.	
Level of first competition:			
CCI*** or CIC***	52.6%	N.A.	
CCI** or CIC**	15.8%	N.A.	
CCI* or CIC*	31.6%	N.A.	
Average interval between competitions (weeks)	3.7 ± 2.8	N.A.	
Range	2–14	N.A.	

All horses and ponies of the national selection were in preparation for the European Championships (EC) in 2010 (ponies) and 2011 (horses), and preparation phase lasted from SET-I (March) towards SET-II (six weeks before EC).

* The number of training weeks before first competition refers to number of weeks between SET-1 at start of competition season and first competition.

N.A.: data not available and CIC, Concours International Combiné’.

CIC; Concours International Combiné; *, **, *** meaning 1-,2- or 3-star eventing level.

### Reasons for withdrawal

Sixteen horses and six ponies were withdrawn before SET-II and the European Championships. The most common reason for withdrawal was locomotor injury, afflicting 56.3% of horses (P < 0.001) and 66.7% of ponies (P = 0.011, Table 2). Horses and ponies did not differ significantly in injury occurrence (odds ratio = 1.44, P = 0.692, 95% CI; 0.20–10.07), and injuries in horses and ponies were not influenced by age or sex. Although there does not seem to be an effect of age on injuries, within ponies and within the horses, the differences of these age effect on injuries (an interaction) was significant (P = 0.007); in horses, the odds for injury increased by 1.87 (95% CI; 0.92–3.81) for each year of age. In ponies, the odds for injury decreased by 2.00 (95% CI; 0.75–5.62) for each year of age.

**Table 2:** Outcome and performance of the national selections in preparation for the European Championship (EC)

	**Horses**	**Ponies**	**Total**
	**n**	**n**	**n**
Total animals	20	9	29
**Withdrawn from EC team**	**16**	**6**	**22**
Injured	9	4	13
*Tendon injury*	*7*	*3*	*10*
*Fetlock-joint injury*	*1*	*-*	*1*
*Lameness (unspecified)*	*1*	*1*	*2*
Did not meet EC competition criteria	4	2	6
Sold	3	-	3
**Participated at EC**	**4**	**3**	**7**
Finished without cross-country faults	2	1	3
Finished with cross-country faults	2	0	2
Eliminated in cross-country	0	2	2

National selection consisted of 20 event horses and 9 event ponies in preparation for European Championships in 2010 (ponies) and 2011 (horses).

### Fitness assessment

Weather conditions were similar during the SET-I tests of horses and ponies (11.9 ± 2.6°C with 72.1 ± 9.3% humidity and 11.1 ± 1.3°C with 68.7 ± 6.8% humidity, respectively). Achieved speeds and HRs of event horses during SET-1 per exercise step were: step 1, 6.5 ± 0.7 m/s and 148 ± 13 beats/min; step 2, 8.9 ± 0.7 m/s and 170 ± 14 beats/min; step 3, 10.4 ± 0.6 m/s and 187 ± 12 beats/min; and step 4, 10.8 ± 0.7 m/s and 196 ± 11 beats/min. Achieved speeds and HRs of event ponies during SET-I per exercise step were: step 1, 8.2 ± 1.2 m/s and 181 ± 11 beats/min; step 2, 9.1 ±0.3 m/s and 188 ± 14 beats/min; step 3, 9.4 ± 0.3 m/s and 201 ± 8 beats/min; and step 4, 9.3 ± 0.4 m/s and 210 ± 8 beats/min. Table 3 presents the fitness indices of horses (n = 17) and ponies (n = 9) for SET-I. Age and sex did not influence fitness in the SET-I of either horses or ponies.

**Table 3:** Fitness indices of event horses and ponies at SET-I at season beginning

	**Horses (n = 17)**	**Ponies (n = 9)**
	**Mean ± sd**	**Mean ± sd**
V_LA2_ (m/s)	9.0 ± 0.7	6.3 ± 0.9
V_LA4_ (m/s)	10.3 ± 0.4	7.8 ± 0.9
HR_LA2_ (beats/min)	175 ± 10	170 ± 17
HR_LA4_ (beats/min)	189 ± 10	180 ± 15
V_200_ (m/s)	11.4 ± 0.8	9.6 ± 0.7
V_170_ (m/s)	8.6 ± 0.8	6.6 ± 0.8
V_140_ (m/s)	5.7 ± 1.2	3.7 ± 1.5
LA_peak_ (mmol/L)	7.6 ± 2.3	13.2 ± 3.1
HR_peak_ (beats/min)	201 ± 7	206 ± 8
V_peak_ (m/s)	10.8 ± 0.7	10.0 ± 0.4
HR_rec5_ (beats/min)	107 ± 19	114 ± 10
HR _rec10_ (beats/min)	88 ± 11	104 ± 10

Seventeen event horses and 9 event ponies of national selection participated in SET-I in preparation for European Championships in 2010 (ponies) and 2011 (horses).

Abbreviations: V_LA2_ and V_LA4_, speed at plasma lactate concentration of 2 and 4 mmol/L, respectively; HR_LA2_ and HR_LA4_, heart rate at plasma lactate concentration of 2 and mmol/L, respectively; LA_peak_, peak plasma lactate concentration; HR_peak_, peak heart rate; V_peak_, peak speed; V_200_, V_170_, and V_140_, heart rate of 200, 170, and 140 beats/min, respectively; HR_rEc5_ and HR_rEc10_, recovery heart rate after 5 and 10 min, respectively; and 3 horses did not participate at SET-I because of injury.

Ambient temperature during SET-IIs of horses and ponies were significantly higher (15.5 ± 2.3°C, P = 0.044) than during SET-I, with relative humidity comparable (64.1 ± 2.9%). LA samples were not taken after the first step of SET-II because, at that stage in SET-I, all horses showed LA below the lowest detectable concentration (0.8 mmol/L).

**Figure 1:**
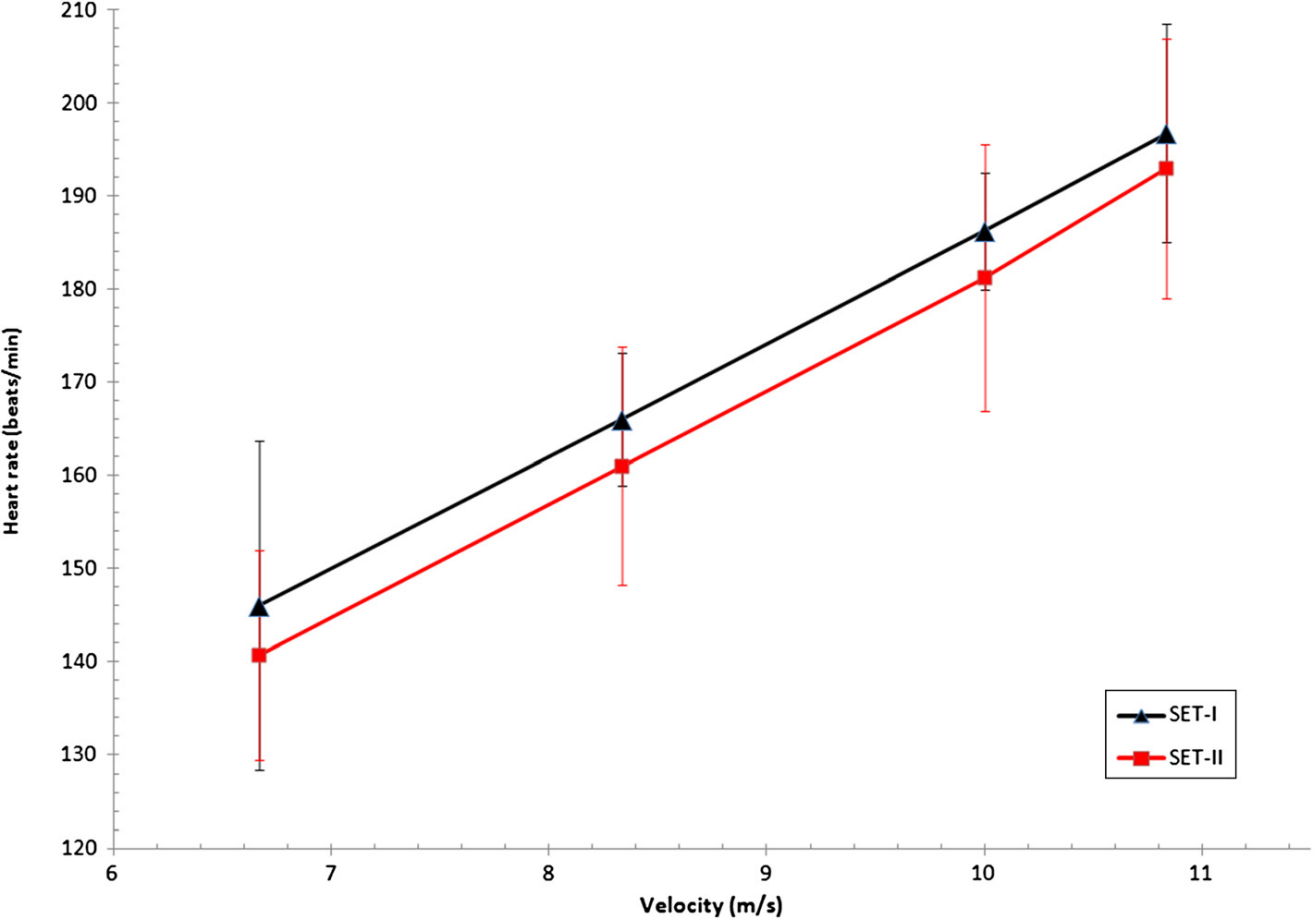
**HR versus velocity of four event horses at both SETs.** HR, heart rate (beats/min); LA, plasma lactate concentration (mmol/L); shows alteration in fitness from SET-I to SET-II of four event horses of national selection in preparation for 2011 European Championships.

**Figure 2:**
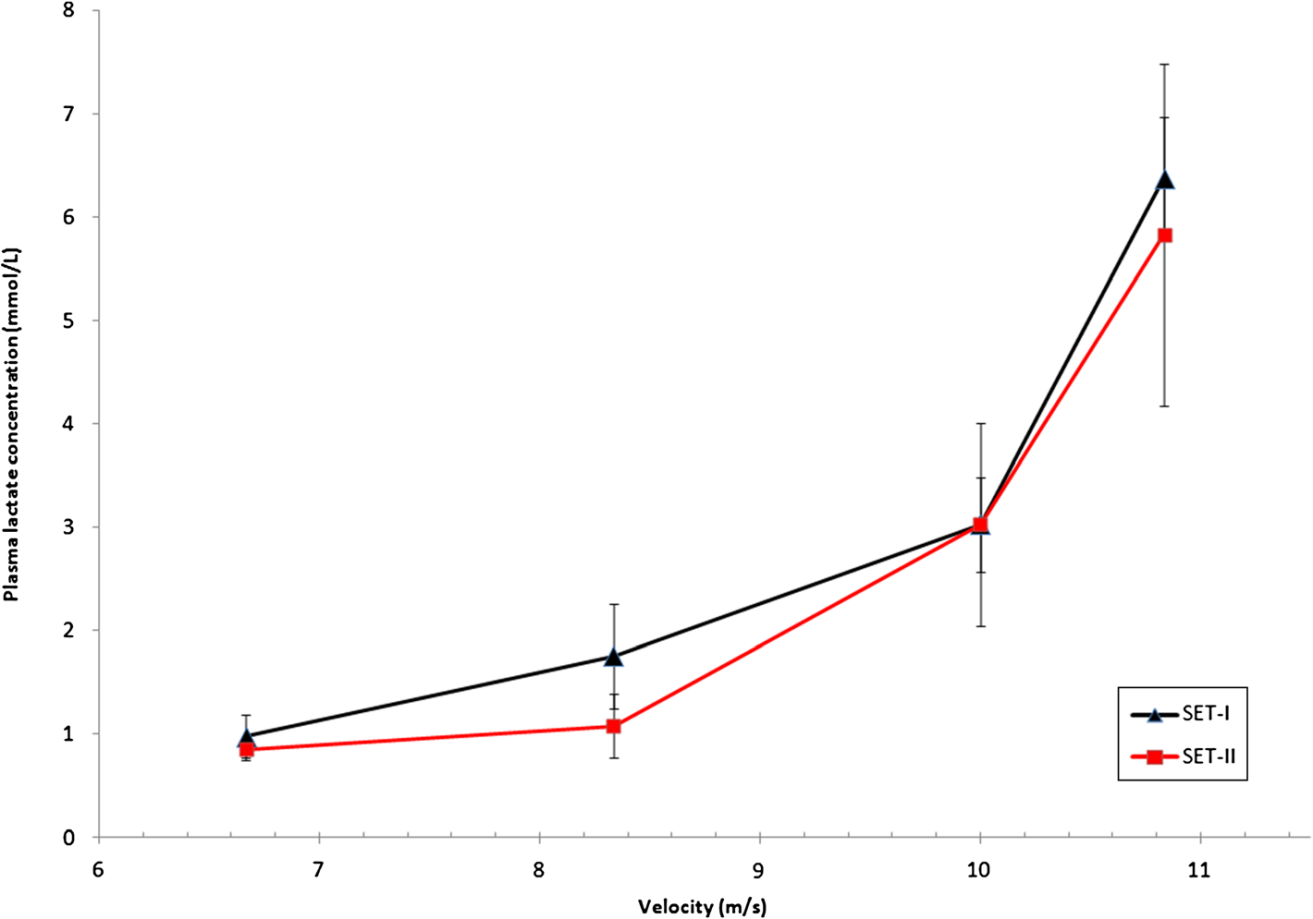
**LA versus velocity of four event horses at both SETs.** HR, heart rate (beats/min); LA, plasma lactate concentration (mmol/L); shows alteration in fitness from SET-I to SET-II of four event horses of national selection in preparation for 2011 European Championships.

**Figure 3:**
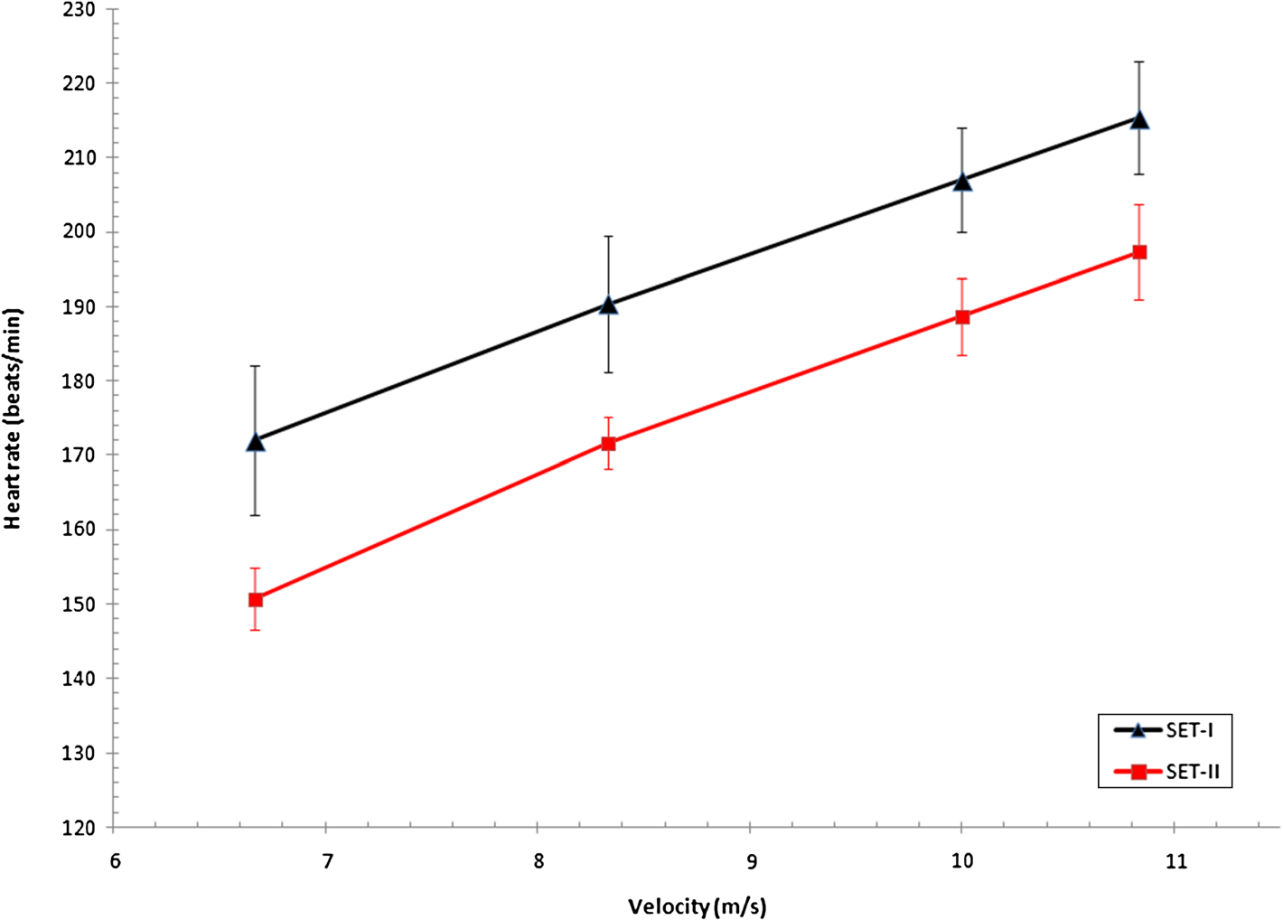
**HR versus velocity of three event ponies at both SETs.** HR, heart rate (beats/min); LA, plasma lactate concentration (mmol/L); shows alteration in fitness from SET-I to SET-II of three event ponies of national selection in preparation for 2010 European Championships.

**Figure 4:**
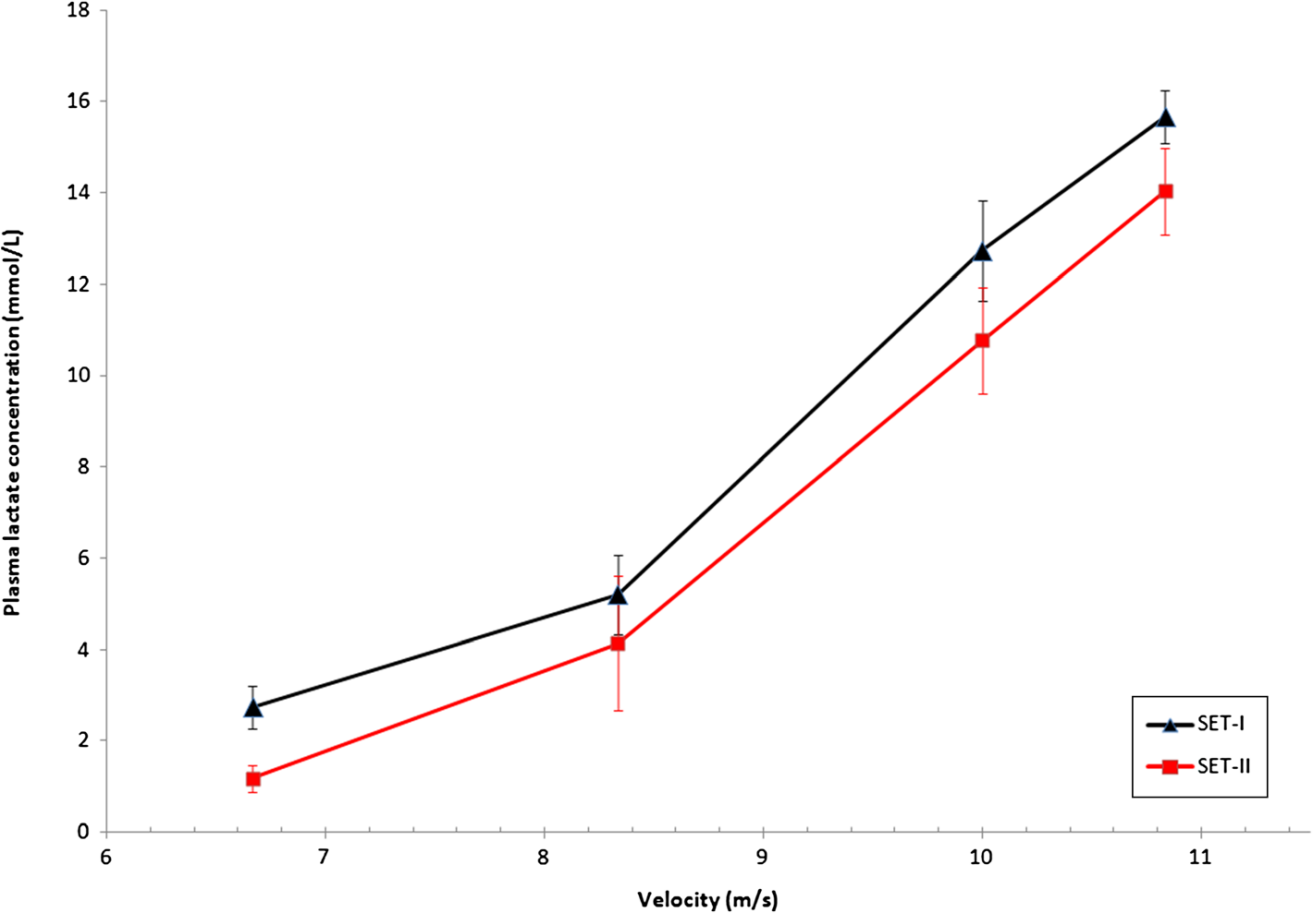
**LA versus velocity of three event ponies at both SETs.** HR, heart rate (beats/min); LA, plasma lactate concentration (mmol/L); shows alteration in fitness from SET-I to SET-II of three event ponies of national selection in preparation for 2010 European Championships.

Four horses and three ponies were able to participate at SET-II (see causes for withdrawal, Table 2). Therefore, achieved speeds and HRs during SET-II for horses and ponies are described in Table 4 and changes in fitness indices described for these horses and ponies in SET-I and SET-II (Table 5, Figures 1,2, 3, and 4).

**Table 4:** Aimed and achieved HRs and speeds in both SETs of event horses and ponies

**Exercise step**	**Aimed speed (m/s)**	**SET-I**		**SET-II**	
		**Achieved speed (m/s)**	**Achieved HR (beats/min)**	**Achieved speed (m/s)**	**Achieved HR (beats/min)**
Horses					
1	6.7	5.7 ± 0.9	146 ± 18	6.4 ± 1.3	141 ± 11
2	8.3	9.0 ± 0.4	166 ± 7	8.6 ± 0.4	161 ± 13
3	10	10.6 ± 0.3	186 ± 6	10.4 ± 0.3	181 ± 14
4	11.7	10.6 ± 0.6	197 ± 12	11.0 ± 0.3	193 ± 14
Ponies	Aimed HR				
	(beats/min)				
1	160	7.6 ± 0.1	172 ± 10	6.8 ± 0.2	151 ± 4
2	170	9.0 ± 0.3	190 ± 9	7.9 ± 0.5	172 ± 4
3	180	9.5 ± 0.4	207 ± 7	8.8 ± 1.3	189 ± 5
4	Max. HR	9.1 ± 0.4	215 ± 8	10.8 ± 0.8	197 ± 6

Aimed and achieved heart rates (HR; beats/min) and speeds (m/s) and achieved HRs speeds during SET-I and SET-II of event horses (n = 4) and event ponies (n = 3) of the national selection in preparation for European Championship of 2010 (ponies) and 2011 (horses).

**Table 5:** Physiological parameters of event horses and ponies at both SETs in preparation for the European Championships

	**Horses (n = 4)**		**Ponies (n = 3)**	
	**Mean ± sd**		**Mean ± sd**	
	*SET-I*	*SET-II*	*SET-I*	*SET-II*
V_LA2_ (m/s)	8.8 ± 1.1	9.3 ± 0.8	6.1 ± 0.7	7.7 ± 0.4
V_LA4_ (m/s)	10.3 ± 0.5	10.3 ± 0.4	7.8 ± 0.3	8.3 ± 0.3
HR_LA2_ (beats/min)	175 ± 10	172 ± 5	167 ± 21	164 ± 0
HR_LA4_ (beats/min)	190 ± 10	184 ± 10	179 ± 16	173 ± 5
V_200_ (m/s)	11.6 ± 1.3	11.7 ± 1.3	9.1 ± 0.7	10.1 ± 0.7
V_170_ (m/s)	8.3 ± 0.4	8.5 ± 0.4	6.3 ± 0.7	7.7 ± 0.9
V_140_ (m/s)	5.0 ± 0.7	6.2 ± 0.2	3.9 ± 1.0	5.4 ± 1.1
LA_peak_ (mmol/L)	7.4 ± 1.8	6.4 ± 1.6	13.0 ± 1.7	16.7 ± 3.2
HR_peak_ (beats/min)	202 ± 4	196 ± 13	212 ± 4	201 ± 8
V_peak_ (m/s)	10.6 ± 0.6	11.5 ± 0.6	9.8 ± 0.5	10.9 ± 0.7
HR _rec5_ (beats/min)	95 ± 7	96 ± 13	103 ± 5	123 ± 39
HR _rec10_ (beats/min)	86 ± 8	83 ± 17	99 ± 12	98 ± 13

Four event horses and three event ponies of the national selection participated in both SETs in preparation for European Championships in 2010 (ponies) and 2011 (horses).

For abbreviation meanings see legend of Table 3.

There was a significant correlation of the V_LA2_ in SET-I and SET-II of each individual in the groups of horses and ponies (r = −0.792, 95% CI −0.97; -0.10). This comparison of SET-I and II indicated that individual horses and ponies improved their V_LA2_ between SETs.

### Fitness and injuries

SET-I data were used to determine the performance groups in the horses and ponies. In SET-I, horse median-reference values were V_LA4_ of 10.3 m/s, HR_rec5_ of 103 beats/min, and HR_rec10_ of 89 beats/min. Among ponies these values were V_LA4_ of 8.0 m/s, HR_rec5_ of 113 beats/min, and HR_rec10_ of 104 beats/min. Based on these values, five event horses and one pony were designated as good performers, and the age and sex of horses and ponies were not different between the performance groups. In addition, horses and ponies were not differently distributed between the performance groups (odds ratio = 0.31, P = 0.38).

Average performers were significantly more likely to be injured (50% of average performers) during the season than good performers (0% of good performers, P = 0.05). In horses, average performing horses were less likely to participate at the European Championship (60% of good performers versus 6.7% of average performers) and in ponies, one good performer and two average performing ponies went to the European Championship.

### Condition training sessions

**Figure 5:**
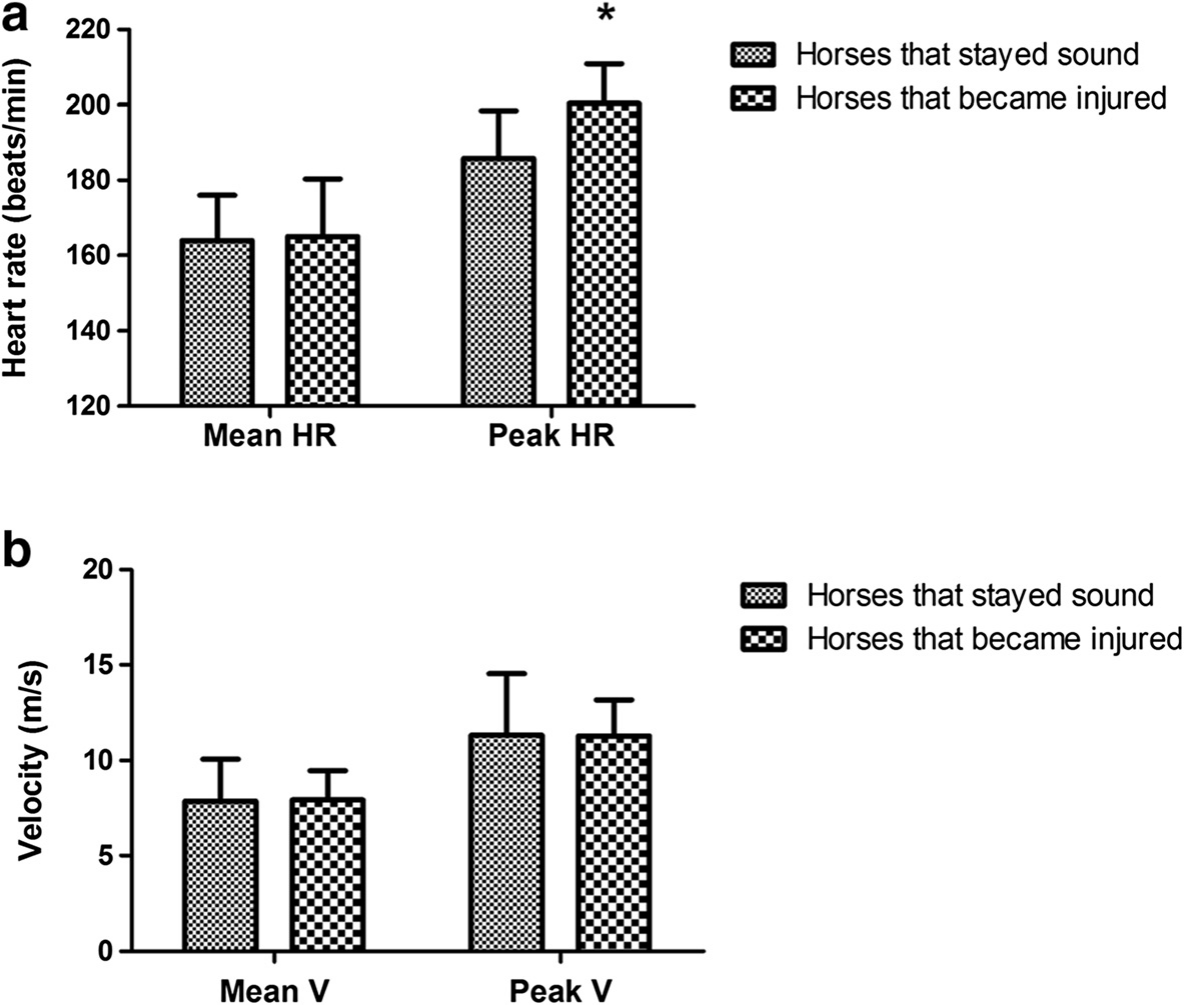
**(a) HR and (b) velocity of training sessions of sound and, later on, injured event horses.** HR, heart rate (beats/min); V, velocity (m/s); subpopulation of ten event horses from national selection in preparation for 2011 European Championships; all conditional training sessions evaluated for HR and velocity; 5 horses became injured (n = 5) and 5 stayed sound (n = 5) during preparation for European Championship; mean and peak HR and V data of condition training sessions analyzed using general linear-model with injury as fixed-effect and horse as random-effect; injury considered effect modifier of data from conditional training sessions; and *, P < 0.001, significantly different compared to horses that stayed sound during preparation for European Championships.

From the subpopulation of ten horses that were followed during condition training sessions, three horses participated at the European Championship, two horses did not meet the competition criteria, and five horses became injured. Table 6 shows the training program of this subpopulation and the means by which physiological parameters were measured. Number, level, and average interval between competitions were not associated with injuries. However, horses that remained sound showed a significantly lower HR_peak_ (186 ± 12 beats/min) during condition training sessions compared with horses that were withdrawn later on from training and competition because of injury (200 ± 10 beats/min; P < 0.001), although peak and mean speed were comparable between these two groups (Figure 5).

**Table 6:** Condition training of event horses from the national selection in preparation for the European Championship

***Condition training sessions in event horses (n = 10)***	**Mean ± sd**
Number of training weeks	9.3 ± 3.9
Training frequency (every …. days)	6.2 ± 2.0
Training/competition ratio	2.7 ± 2.2
Condition training sessions	
Number of training bouts	2.5 ± 1.3
Duration training bout (min)	5.9 ± 1.5
Mean heart rate training bout (beats/min)	165 ± 7
Mean speed training bout (m/s)	7.1 ± 2.6
Maximum heart rate training bout (beats/min)	193 ± 11
Maximum speed training bout (m/s)	10.3 ± 3.8

In total, 62 condition training sessions registered (ranging from 3 to 14 per horse) and number of training bouts defined as ‘separate galloping sets within one training session, alternated with resting periods in between each training bout’.

## Discussion

The present study was not based on large numbers of subjects, which is admittedly a weakness. However, this is the first study that prospectively monitors, over a prolonged period, the fate and performance of entire national horse and pony teams during preparations for a major event, in this case the European Championship. This unique experimental set-up provided insight into the reasons for withdrawal, fitness, and injury incidence in a cohort of event horses and ponies competing at a high level.

An interesting observation here was that 45% of the national selection of horses and ponies had to be withdrawn because of locomotor injury during preparations for the European Championship. The results of a training study in the United Kingdom shows that 21% of horses intended to compete in a CCI did not start because of an injury [2]. Additionally, in another study, also performed in the United Kingdom, evaluation of wastage in event horses showed that 35.1% of these horses (n = 2138) were not re-registered the next year because of veterinary problems [1]. These published values are somewhat lower than results in the present study but still in the same order of magnitude. The higher percentage of injuries here might simply have been due to lower numbers and random variation but might also have been related to the fact that all animals competed at the top level, yielding them more prone to injuries. There is urgent need, with the goal of reducing injury incidence, for similar information concerning event animals in other countries to establish whether such high wastage percentages are universal and to better understand the effects of training methods.

When comparing fitness levels of the event horses and ponies in the present study with literature data which involved similar SET protocols, but keeping in mind the restrictions of field exercise testing, the present horses performed better (V_LA4_ = 10.3 ± 0.4; HR_4_ = 189 ± 10; V_200_ = 11.4 ± 0.8) and, therefore, possessed a higher level of fitness than Spanish three-day event horses that had undergone minimal training (V_LA4_ = 8.3 ± 0.5; HR_4_ = 163 ± 3) [6], Belgian event horses competing at national level (V_LA4_ = 9.2; [3], V_LA4_ = 8.6 ± 0.2; V_200_ = 10.8 ± 0.3) [5], and Swiss event horses up to CCI** level (V_LA4_ = 8.5 ± 0.9; HR_4_ = 185 ± 10_;_ V_200_ = 9.6 ± 0.8) [4]. To the authors’ knowledge, there is no information available on event ponies, but ponies from the current study had slightly higher V_LA4_ values (7.8 ± 0.9) than research ponies after a moderate or strenuous training period (7.2 ± 1.2) [12]. Differences between horses and ponies can be explained by their different ventilatory responses to exercise [21,22] and/or their dissimilar metabolic demands at competition.

Two horses and eight ponies had V_LA4_ values at SET-I below the average speed demanded at the level of the European Championships (for horses the acquired average speed is 9.5 m/s and for ponies 8.7 m/s). These animals should be considered unfit to compete at European Championship level because an increase in workload above the anaerobic threshold results in an exponential rise in LA, leading to rapid onset of fatigue [23]. This phenomenon has also been described by Amory et al. [3] in their study and by Serrano et al. [24], who concluded that if animals are less fit, their performance with be compromised, and the risk of non-completion injury will be increased. In the present study, equine athletes with better performance parameters were less likely to get injured than average performers. The field test used here showed predictive value for subsequent injuries in event horses and ponies and, thus, might aid in assessing risk factors and minimize injuries in the future.

As event horses age, the odds of injury increase, which is in accordance with data found in racehorses and British dressage horses [25,26]. It has been suggested that accumulated microdamage or degenerative changes associated with age and increasing training and/or competition level might predispose them to injury. However, an opposite relation was found here in ponies. The reason for this is not entirely clear, but this might have been due to the low number of ponies; there is also a possibility that these young event ponies were more easily overtrained/injured than young event horses because most pony riders are less experienced. This latter possibility is supported by the fact that horse riders were able to ride both SETs at comparable work intensities and pony riders were not able to do so. The present pony riders had difficulties riding at predetermined HRs, as most pony riders rode too fast too soon in SET-I, despite very clear instructions at which HR they should ride during the SET. In addition, ‘each pony’s individual maximal heart rate’ might not have actually resulted in that horse obtaining their peak heart rate as rider variation in pushing the animals needed to be taken into account. This topic requires further investigation.

Recovery HR and V_LA4_ have been shown to be good indicators of fitness and performance [4,14,27]. However, in the literature, several methods, with cut-off values unreported or differing among these studies, have been used to discriminate between good and average performers. It would be easier if, in the future, similar methods and cut-off values were used to separate average and good performing event horses.

Heart rate and other physiological parameters of horses might increase with increasing ambient temperatures [28]. Even if the physiological variables had been the same in SET-I and SET-II, the results indicated better fitness as environmental temperature increased. The fact that the physiological variables were lower in SET-II suggested an even greater improvement in fitness but, as described above, the numbers were too low for further statistical evaluation. Fitness improvement appeared more pronounced in the ponies but, although this appeared a plausible conclusion, the effect might also have been due to the use of a different racetrack in SET-I [14]. The use of different racetracks for the exercise tests in ponies might have influenced the ponies’ physiological parameters as different track qualities might have influenced the workload. Research has shown that horses have higher heart rates when working in deep sand than on firm surfaces [29]. This might also explain the fact that the work intensity of the first three exercise steps of SET-I in ponies was somewhat higher compared with SET-II.

Horses of the subpopulation were trained at a much lower speed than required during competition; similar observations have been made by Serrano et al. [24] and Baumann et al. [30]. An explanation for this might be that riders believe that (heavy) condition training increases the risk of injuries. However, insufficiently trained horses might not be physiologically adapted to the high demands of competition [24]. In addition, HR measured here during condition training appeared to have predictive value for pending injuries, which is an interesting observation that, to the knowledge of the authors, has not been previously described, but one that also needs confirmation in a larger population. It was assumed that the increased heart rate observed here was an effect of the early stages of lameness, not yet detected by the rider or trainer, which seems to precede visible lameness in a horse.

## Conclusions

There was a high wastage percentage in the Dutch national selections of event horses and ponies training for a major event. Most animals were withdrawn from the competition because of locomotor injuries.

To prevent injuries, it is important that event horses and ponies are sufficiently fit to accommodate the physiological demands necessary for high-level competition. This study provides preliminary evidence (requiring confirmation in larger populations) that careful monitoring of the horses might assist in realizing this goal.

Field tests were useful in assessing the potential injury risk, as individuals with better fitness indices (good performers) were less likely to be injured than average performers. Further, monitoring of training sessions showed predictive value for future injuries, as horses that became injured later on had increased peak HR during condition training than horses that remained sound. The increase in peak HR seemed to precede visible lameness in a horse.

## Endnotes

^1^See: http://www.fei.org.

^2^See: http://www.knhs.nl.

## Competing interests

This research was partly supported by a grant of the Dutch National Equestrian Federation (KNHS). No conflicts of interest have been declared.

## Authors’ contributions

CM designed and carried out the cohort study and drafted the manuscript. JB contributed to the study design, data analysis and interpretation, and manuscript revision. EW contributed to data acquisition and manuscript revision. RW helped in study design, data interpretation, and critically revised the manuscript. MS participated in the study and manuscript desing, data interpretation, and helped draft and critically revise the manuscript. All authors read and approved the final manuscript.
